# De Novo Assembly, Functional Annotation and Comparative Analysis of *Withania somnifera* Leaf and Root Transcriptomes to Identify Putative Genes Involved in the Withanolides Biosynthesis

**DOI:** 10.1371/journal.pone.0062714

**Published:** 2013-05-08

**Authors:** Parul Gupta, Ridhi Goel, Sumya Pathak, Apeksha Srivastava, Surya Pratap Singh, Rajender Singh Sangwan, Mehar Hasan Asif, Prabodh Kumar Trivedi

**Affiliations:** 1 National Botanical Research Institute, Council of Scientific and Industrial Research (CSIR-NBRI), Rana Pratap Marg, Lucknow, India; 2 Central Institute of Medicinal and Aromatic Plants, Council of Scientific and Industrial Research (CSIR-CIMAP), Lucknow, India; 3 Department of Biochemistry, Faculty of Science, Banaras Hindu University, Varanasi, India; Nanjing Agricultural University, China

## Abstract

*Withania somnifera* is one of the most valuable medicinal plants used in Ayurvedic and other indigenous medicine systems due to bioactive molecules known as withanolides. As genomic information regarding this plant is very limited, little information is available about biosynthesis of withanolides. To facilitate the basic understanding about the withanolide biosynthesis pathways, we performed transcriptome sequencing for *Withania* leaf (101L) and root (101R) which specifically synthesize withaferin A and withanolide A, respectively. Pyrosequencing yielded 8,34,068 and 7,21,755 reads which got assembled into 89,548 and 1,14,814 unique sequences from 101L and 101R, respectively. A total of 47,885 (101L) and 54,123 (101R) could be annotated using TAIR10, NR, tomato and potato databases. Gene Ontology and KEGG analyses provided a detailed view of all the enzymes involved in withanolide backbone synthesis. Our analysis identified members of cytochrome P450, glycosyltransferase and methyltransferase gene families with unique presence or differential expression in leaf and root and might be involved in synthesis of tissue-specific withanolides. We also detected simple sequence repeats (SSRs) in transcriptome data for use in future genetic studies. Comprehensive sequence resource developed for *Withania*, in this study, will help to elucidate biosynthetic pathway for tissue-specific synthesis of secondary plant products in non-model plant organisms as well as will be helpful in developing strategies for enhanced biosynthesis of withanolides through biotechnological approaches.

## Introduction

*Withania somnifera* popularly known as Ashwagandha or Indian ginseng is a small woody shrub of Solanaceae family. The plant is one of the most reputed medicinal herbs that form an essential constituent of Ayurvedic and indigenous medical system [1]. Historically, the plant has been used as an aphrodisiac, liver tonic, immunoprophylactic, anti-inflammatory agent, astringent, and more recently to treat bronchitis, asthma, ulcers, emaciation, insomnia, and senile dementia [2], [3], [4]. Clinical trials and animal research support pharmacological activities of *Withania* for cognitive and neurological disorders, inflammation, stress, Parkinson’s and Alzheimer’s diseases, intestinal disorders, immunomodulation, hepatoprotection, etc. [3], [5], [6], [7]. Chemo preventive properties of *Withania* suggest this herb as a potentially useful adjunct for patients undergoing radiation and chemotherapy [8], [9].

The medicinal properties of *W. somnifera* have been correlated with withanolides and their glycoconjugates (glycowithanolides) present in various plant parts [3], [10]. Withanolides, a group of naturally occurring secondary metabolites, are generally C-28 steroidal lactones in which C-22 and C-26 are oxidized to form lactone ring [11]. Various chemotypes of *Withania* accumulating more than 40 withanolides and several sitoindosides in aerial parts, roots and berries have been reported [12], [13], [14]. Studies suggest that precursor molecules for withanolide biosynthesis are isoprenoids. These isoprenoids are synthesized via classical cytosolic mevalonate (MVA) pathway and plastid localized 2-C-methyl-D-erythritol-4-phosphate (MEP) pathway leading to biosynthesis of 24-methylene cholesterol (C_30_ terpenoid) [15], [16]. This central molecule through various biochemical reactions including hydroxylation and glycosylation leads to the production of various withanolides [17]. Different withanolides have been shown to be present in various plant parts, however, a few show tissue-specific biosynthesis. It has been demonstrated that withaferin A is mainly synthesized in leaf tissue whereas withanolide A is synthesized specifically in root tissue [11], [12], [14]. Though various studies have been carried out to establish metabolic profiles and pharmacological activities of *Withania*, very limited information is available on biosynthetic pathways of withanolides, from this important medicinal plant. Recently, biochemical and molecular studies have been initiated which led to characterize few genes/enzymes from this important plant [11], [17], [18], [19], [20], [21], [22].

Despite pharmacological importance, the transcriptomic and genomic data of *W. somnifera* are very limited and only 742 ESTs are available in the National Center for Biotechnology Information (NCBI) database. This limited information hinders the study of withanolide biosynthetic pathway in *Withania*. Due to immense importance, this plant has been selected under sequencing of 100 Solanaceae genomes by Sol Genomics Network (http://solgenomics.net/organism/sol100/view). Studies suggests that generation and analysis of Expressed Sequence Tags (ESTs) can be a useful tool for the purposes of gene discovery especially in non-model plants for which no reference genome sequences are available [23]. The in-depth generation of EST databases of *Withania* may provide information about all the expressed regions of genome and can be used to characterize patterns of gene expression in specific tissues. Recently, using Next-Generation Sequencing (NGS) such databases have been developed and used for discovery and prediction of genes involved in secondary metabolite biosynthesis from medicinally important plants [24], [25], [26]. These databases are also a rich source of gene-derived molecular markers (e.g. simple sequence repeat, SSR) which can be used for germplasm breeding or physical mapping [27].

Our main objective of this study is to establish the basic understanding about the withanolide biosynthesis pathway operating in root and leaf tissues. We established transcriptome data of leaf and root tissues of *Withania* (NMITLI-101 chemotype) using NGS technology based on 454 GS FLX Titanium platform and identified genes which may be involved in withanolide biosynthesis pathway. In this study, we report EST collection of leaf and root from *Withania* with a number of differentially expressed methyltransferases, cytochrome P450s, glycosyltransferase and transcription factors which may be involved in differential withanolide biosynthesis in leaf and root. Using transcriptome data, we also analyzed molecular markers of EST-SSRs for facilitation the marker-assisted breeding of this species.

## Materials and Methods

### Plant Material, cDNA Library Construction and Sequencing

Leaf and root tissues of *W. somnifera* (chemotype NMITLI-101*)* were collected from one-year old field grown plants from experimental plot in the CSIR-National Botanical Research Institute (Lucknow) garden. Frozen tissues were ground to a fine powder in liquid nitrogen and total RNA was extracted using Spectrum Plant Total RNA Kit (Sigma–Aldrich, USA) and treated with RNase free DNaseI (Ambion, USA) according to manufacturer’s instructions. The quality and quantity of total RNA were analyzed by agarose gel and spectrophotometric analysis (ND-1000 Nanodrop, NanoDrop Technologies, USA). The equal amount of total RNA from four different preparations was pooled and used for further processing. Double-stranded (ds) cDNA library was prepared using pooled total RNA and double stranded cDNA synthesis kit (Invitrogen, Carlsbad, CA) as per manufactures recommendations. Quantity as well as quality of (ds) cDNA library was checked on Agilent 2100 Bioanalyzer DNA chip (Agilent Technologies Inc., Santa Clara, CA). (ds) cDNA was fragmented by nebulization to produce random fragments of about 250–800 bp in length and purified using QIAquick PCR purification spin columns (Qiagen, USA). Smaller fragments (below 300 bp) were removed and purified cDNA samples were assessed on DNA chip (Agilent 2100 Bioanalyzer, USA). Adapter ligation and purification of adapter ligated (ds) cDNA library was done according to manufacturer’s instruction (Roche, USA). (ds) cDNA fragments were denatured to generate single-stranded cDNA fragments, which were further amplified by emulsion PCR for sequencing according to manufacturer’s instructions (Roche, USA). The sequencing of leaf and root cDNA libraries was performed on a 454-GS FLX sequencing platform (454 Life Sciences, Roche, USA) using GS FLX Titanium Kit.

### *Denovo* Assembly and Sequence Annotation

The 454 raw read sequences from leaf and root libraries were used for quality-filtering algorithms and filtered for weak signals, low-quality sequences, and the read ends were screened and trimmed for 454 adaptor sequences to yield high quality (HQ) sequences (>99.5% accuracy on single base reads). The data from the 454 read sequences were assembled into unique sequences using ROCHE GS Assembler (version 2.5.3) with 40 base pair overlap and 95% identity. Because no reference genome exists for *Withania*, reads were assembled *de novo*. The contigs and singletons of leaf and root libraries (101L and 101R) were annotated using standalone version of BLASTx program against Arabidopsis protein database at The Arabidopsis Information Resource (TAIR; http://www.arabidopsis.orgTair10), the NCBI non-redundant protein (Nr) database (http://www.ncbi.nlm.nih.gov; released on 06/04/2012), potato (http://solgenomics.net/organism/Solanum_tuberosum/genome; v3.4) and tomato (http://solgenomics.net/organism/Solanum_lycopersicum/genome; version ITAG2.3) with an E-value cut-off of 10^−5^ and extracting only the top hit for each sequence.

### Functional Characterization and Biological Pathways Assignment

To assign function to each unigene, gene ontology (GO) analysis was performed using GO annotation in online TAIR Database (http://www.arabidopsis.org/), which classified Unigenes under the categories of Cellular component, Molecular Function and Biological Process. The TAIR IDs of all the unigenes (contigs and singletons) from leaf and root were retrieved from TAIR10 annotation. Each annotated sequence may have more than one GO term, either assigned in the different GO categories (Biological Process, Molecular Function and Cellular Component) or in the same category [28]. To gain an overview of gene pathway networks, the assignment of polypeptides encoded by unigenes from root and leaf transcriptome into metabolic pathways were mapped according to the Kyoto Encyclopedia of Genes and Genomes (KEGG) [29]. Enzyme commission (EC) numbers were assigned to unique sequences, based on the BLASTx search of protein databases, using a cutoff E value 10^−5^. The output of KEGG analysis includes KEGG orthology (KO) assignments using KEGG automated annotation server, KAAS (http://www.genome.jp/kaas-bin/kaas_main?mode=partial).

### Digital Gene Expression Profiling

To study digital gene expression of unigenes between leaf and root tissues, the log2 converted values for each contig were subjected in MeV (version 4.8.1). To carry out this, the contigs and singletons of leaf and root libraries (101L and 101R) were tagged, assembled together and annotated. Transcripts per million (tpm) were calculated for each contig formed for further analysis. The total unigenes formed were annotated again using standalone version of BLASTx program against protein at The Arabidopsis Information Resource (TAIR; http://www.arabidopsis.orgTair10), the NCBI non-redundant protein (Nr) database (http://www.ncbi.nlm.nih.gov; released on 06/04/2012), potato (http://solgenomics.net/organism/Solanum_tuberosum/genome; v3.4) and tomato (http://solgenomics.net/organism/Solanum_lycopersicum/genome; version ITAG2.3) with an E-value cut-off of 10^−5^ and extracting only the top hit for each sequence. The digital gene expression profiling was carried out for biosynthesis of terpenoid backbone, cytochrome P450s, glycosyltransferases and methyltransferases to generate heat map after annotation and identification of these gene families. Differential expression of genes was calculated by using R value and fold change. Log2 converted values for each contigs were subjected in MeV (version 4.8.1). If the R value ≥8 and fold change ≥2 then a gene was considered as differential. Previously, similar analysis was used for identification of differentially expressed genes from different data sets [30]. Hierarchical clustering (HCl) of log-transformed expression data was carried out using Euclidean Distance Matrix and Average linkage clustering method [31].

### Real-time PCR Analysis

Expression of selected differentially expressed cytochrome P450s was analyzed through qRT-PCR using Real-Time PCR Detection System (ABI 7500, Applied Biosystems, USA) and fast SYBR Green PCR Master Mix (Applied Biosystems, Warrington, UK) to validate 454 sequencing data. Each PCR reaction was set up in total 20 µl volume containing 10 µl of Fast SYBR green master mix, 10 ng of cDNA sample prepared using RevertAid H minus first strand cDNA synthesis Kit (Fermantas, Life Sciences, Ontario, Canada) and gene-specific primers (Table S1). The PCR cycling conditions were as follows: 50°C for 2 min, 95°C for 2 min for initial denaturation followed by 40 cycles of 95°C for 15 s and 60°C for 60 s. Each time, reaction sets also incorporated a no-template control. We employed probes specific for the actin as references. The qPCR data was analysed with the ΔΔCT method using reference gene. For each sample, the mRNA levels of target genes were normalized to that of the actin mRNA. All the experiments were repeated using three biological replicates and the data were analyzed statistically (± Standard Deviation).

### Simple Sequence Repeats (SSRs) Identification

All the contigs and singletons of leaf and root assemblies were used in a microsatellite program (MISA) (http://pgrc.ipk-gatersleben.de/misa/misa.html) for identification of SSR motifs. We searched for microsatellites from mononucleotide to hexa nucleotide. The parameters used for simple sequence repeats (SSRs) were at least 6 repeats for di- and 5 for tri-, tetra, penta- and hexa- nucleotide. Both perfect (i.e. contain a single repeat motif like such as ‘ATC’) and compound repeats (i.e. composed of two or more motifs separated by 100 bases) were identified.

## Results

### Transcriptome Sequencing, Assembly and Annotation

**Figure 1:**
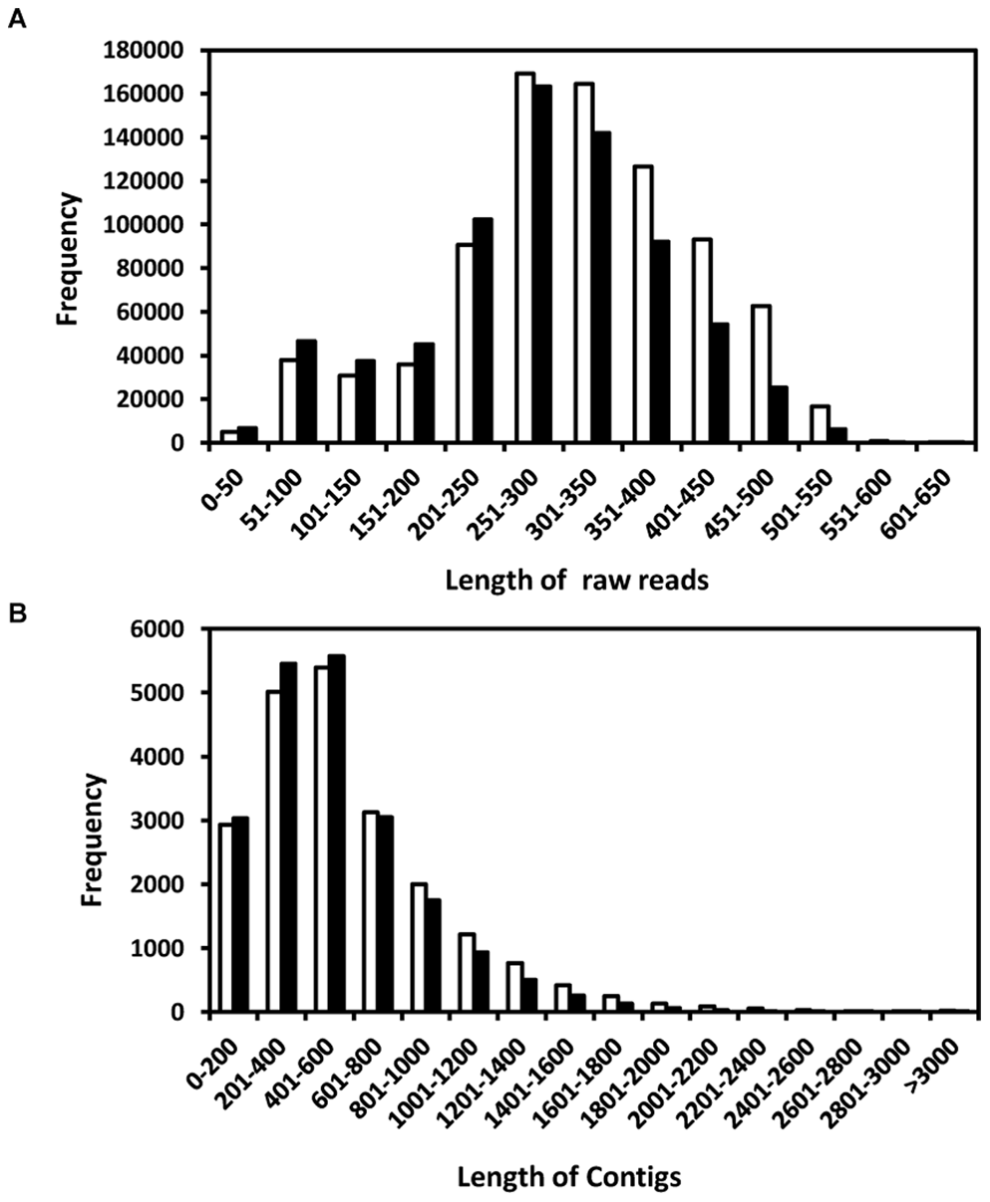
Size distribution of 454 high-quality reads (A) and assembled contigs (B). Black and white bars represents reads and contigs from leaf (101L) and root (101R) transcriptome data respectively.

cDNA libraries from leaf and root tissues of *W. somnifera* (NMITLI-101) were sequenced using half plate run for each on a 454-GS FLX Titanium platform. Sequencing run yielded 8,34,068 (260 Mb) and 7,21,755 (203 Mb) high-quality (HQ) expressed sequence tags for 101L and 101R respectively (Table 1). The sequences from both the libraries were deposited at NCBIs Short Read Archive under the accession number SRA053485 (leaf run no. SRR520142 and root run no. SRR520143). Approximately 90% of the reads obtained were between 200 and 600 bp from both libraries. From 101L library, 6,40,398 reads were assembled into 21,445 contigs and 68,103 reads remained as singletons. Similarly, from 101R library, 5,03,970 reads were assembled into 20,797 contigs and 94,017 singletons. Average length of contigs in 101L and 101R was 590.75 bases (range 100–5001 bases) and 533.15 bases (100–6724 bases) respectively (Table 1). The size distribution of the raw reads and assembled contigs is shown in Fig. 1. Among all the contigs, 49.6% contigs from 101L and 44.6% contigs from 101R were considered as large contigs with nucleotide length more than 500 bp. GC content of contigs of 101L and 101R was 41.07% and 40.48% respectively, whereas in singletons, GC content of 39.10% and 38.19% was observed in 101L and 101R respectively (Table 1).

**Table 1:** Summary of 454 sequencing and assembly for leaf (101L) and root (101R) of *Withania somnifera.*

Features	101L	101R
HQ reads	834068(259581875 bp)	721755(203441421 bp)
Average HQ read length	311.63 bp	282.28 bp
Reads Assembled	640398	503970
Number of contigs	21445	20797
Average length of contigs	590.75 bp	533.15 bp
Range of contigs length	100–5001 bp	100–6724 bp
Contigs above 200 bp	18515 (86.34%)	17765 (85.63%)
GC content	41.07%	40.48%
Percentage of large contigs	49.6%	44.6%
Average length of large contigs	885 bp	821 bp
Number of Singletons	68103	94017
Average length of singletons	280.64 bp	259.61 bp
Range of singleton lengths	50–602 bp	50–612 bp
Singletons above 200 bp	53115 (77.99%)	69451 (73.87%)
GC content	39.10%	38.19%

**Figure 2:**
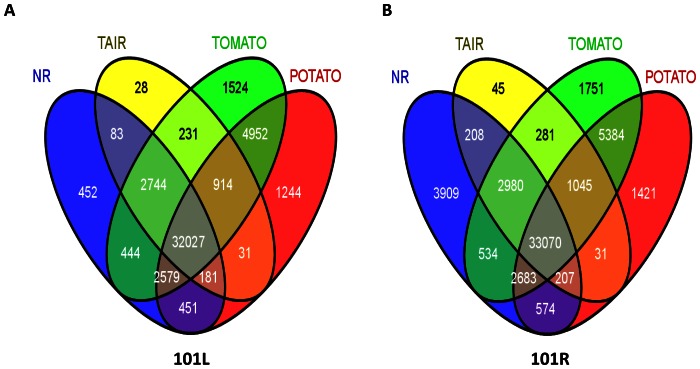
The number of unigenes of 101L (A) and 101R (B) transcriptomes annotated using TAIR10, NR, tomato and potato databases.

BLAST search results of assembled sequences of 101L and 101R against the NR, TAIR10, tomato and potato genome databases, including the species name, accession number and E-value, are presented in Tables S2, S3, S4, S5, S6, S7, S8 and S9. BLAST annotation of large number of contigs and singleton sequences indicated extensive coverage of the *W. somnifera* transcriptome (Table 2). In 101L, 36,239 and 38,961 unigenes were annotated in TAIR10 and NR databases respectively. However, annotation using tomato and potato databases was much higher in comparison to TAIR10 and NR (Table 2). Using tomato and potato databases, a total of 54,639 and 54,462 unigenes could be annotated. Out of these, 32,027 unigenes were annotated common through TAIR10, NR, tomato and potato databases. 452, 28, 1524 and 1244 unigenes annotated uniquely in NR, TAIR10 tomato and potato databases respectively (Fig. 2A). In case of 101R, 37,867, 44,165, 55,415 and 47,677 unigenes got annotated against TAIR9, NR, tomato and potato databases respectively. Number of common unigenes which got annotation from all the databases was 33,070, 3909, 45, 1751 and 1421 unigenes annotated uniquely in NR, TAIR10 tomato and potato databases respectively (Fig. 2B).

**Table 2:** Annotation details of 101L and 101R transcriptomes using various databases.

	101L		101R	
	Contigs	Singletons	Contigs	Singletons
**Total**	21,445	68,103	20,797	94,017
**TAIR10**	14,548	21,691	12,970	24,897
**NCBI (nr)**	15,240	23,721	13,975	30,190
**Tomato**	18,138	36,501	16,477	38,938
**Potato**	19,351	35,111	14,626	33,051

### Gene Ontology Classification

**Figure 3:**
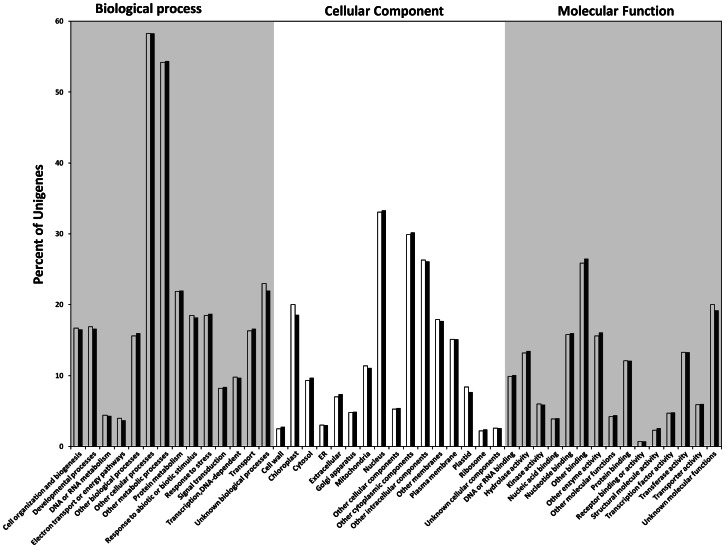
Histogram of gene ontology classification. The results are summarized in three main categories: biological process, cellular component and molecular function. Bars represent the percent number of assignments of leaf (101L; white bars) and root (101R; black bars) unigenes with BLAST matches in the TAIR10 database to each GO term.

GO assignments were used to classify the functions of the 101L and 101R unigenes. Based on sequence homology, 36,239 sequences from 101L and 37,867 sequences from 101R were categorized into 45 functional groups (Fig. 3; Table S10). In each of the three main categories (biological process, cellular component and molecular function) of the GO classification. ‘other Cellular process’ and ‘other metabolic process’ terms are dominant (>50%) respectively. High percentage of genes from categories of ‘nucleus’, ’other cytoplasmic components', ‘other binding’ and ‘other intracellular components' were classified. Extremely low percentage of genes were classified in terms of ‘receptor binding or activity’ category (Fig. 3). Both the libraries showed similar type of distribution pattern of unigenes under different GO terms. A large number of unigenes from 101L and 101R were designated to ‘other metabolic processes’, which suggests that our study may allow for the identification of novel genes involved in the secondary metabolite biosynthesis pathways from root and leaf tissues of *W. somnifera*.

### Functional Characterization Using KEGG

Functional classification and pathway assignment was performed by the Kyoto Encyclopedia of Genes and Genomes (KEGG). To identify the biological pathways that are functional in the leaf and root tissues of *W. somnifera*, 36,239 and 37,867 annotated sequences from 101L and 101R respectively were mapped to the reference canonical pathways in KEGG. In total, all contigs and singleton sequences from 101L and 101R were assigned to 124 KEGG pathways. Interestingly, 1,068 and 1,034 unigenes from 101L and 101R and a total 1,500 unigenes were found involve in biosynthesis of various secondary metabolites (Table 3). Out of all secondary metabolite pathways, the cluster for ‘Phenylpropanoid biosynthesis [PATH: ko00940]’ represents the largest group (292 members) followed by ‘Terpenoid backbone biosynthesis [PATH: ko00900]’ (170 members) and ‘Limonene and pinene degradation [PATH: ko00903]’ (120 members).

**Table 3:** The unigenes related to secondary metabolites in 101L and 101R.

Secondary metabolites biosynthesis pathways	Unigenes in 101L	Unigenes in 101R	Total Unigenes
Anthocyanin biosynthesis [PATH: 00942]	5	3	5
Brassinosteroid biosynthesis [PATH: 00905]	15	17	25
Caffeine metabolism [PATH: 00232]	15	14	18
Carotenoid biosynthesis [PATH: 00906]	80	80	117
Cutin, suberin and wax biosynthesis [PATH: 00073]	10	18	26
Diterpenoid biosynthesis [PATH: 00904]	25	24	33
Flavone and flavonol biosynthesis [PATH: 00944]	43	19	45
Flavonoid biosynthesis [PATH: 00941]	48	64	76
Indole alkaloid biosynthesis [PATH: 00901]	8	5	11
Isoquinoline alkaloid biosynthesis [PATH: 00950]	42	39	58
Limonine and pinene degradation [PATH: 00903]	92	75	120
Monoterpenoid biosynthesis [PATH: 00902]	9	9	11
Nicotinate and nicotinamide metabolism [PATH: 00760]	36	37	48
Phenylpropanoid metabolism [PATH: 00940]	192	206	292
Sesquiterpenoid and triterpenoid biosynthesis [PATH: 00909]	50	36	69
Steroid biosynthesis [PATH: 000100]	54	50	67
Stibenoid diarylhepatanoid and gingerol biosynthesis [PATH: 00945]	82	79	112
Terpenoid backbone biosynthesis [PATH: 00900]	124	112	170
Tropane, piperidine and pyridine alkaloid biosynthesis [PATH: 00960]	39	55	62
Ubiquinone and other terpenoid-quinone biosynthesis [PATH: 00130]	73	70	101
Zeatin biosynthesis [PATH: 00908]	26	22	34
**Total**	**1068**	**1034**	**1500**

### Features of Combined Assembly

**Figure 4:**
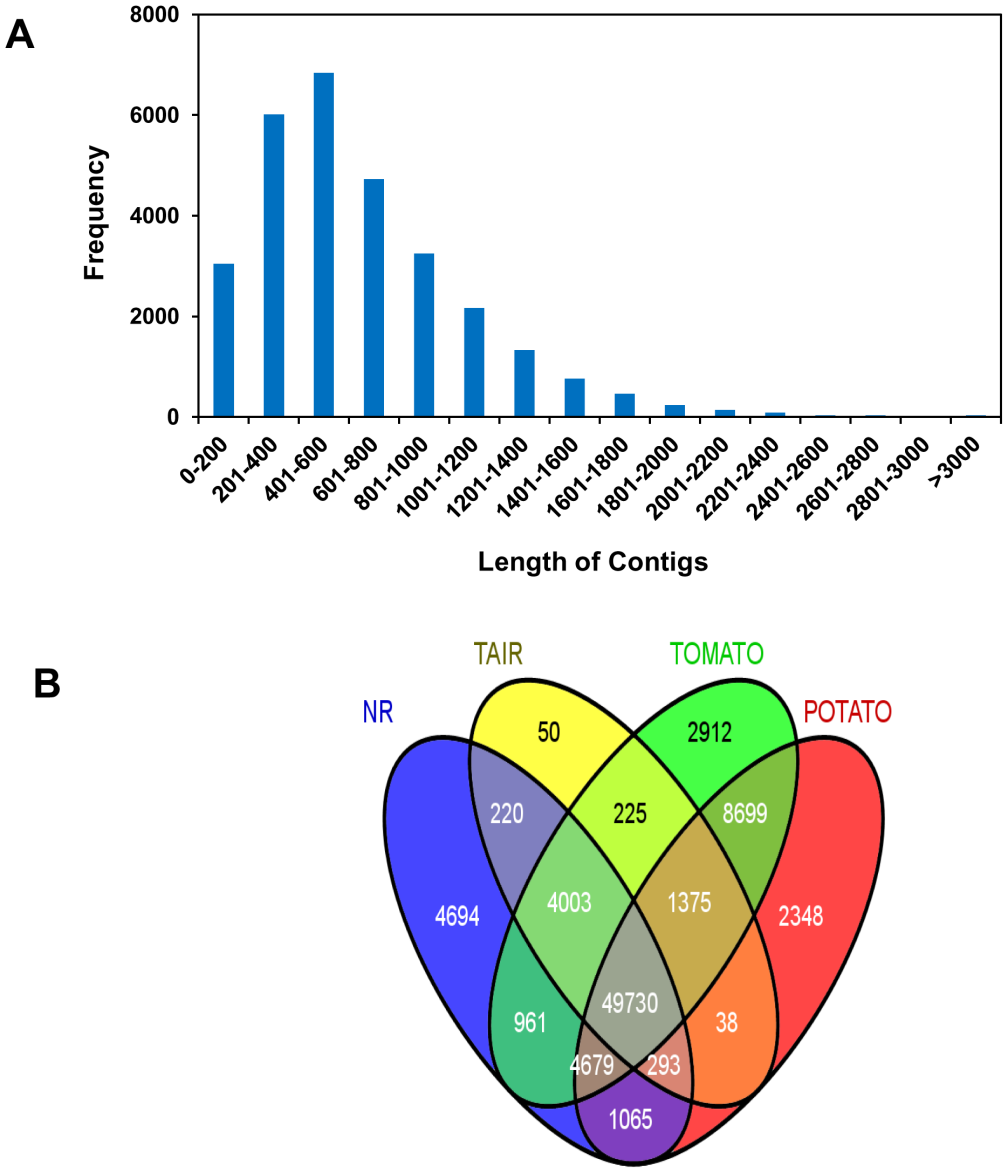
Size distribution of assembled contigs (A) and annotation (B). Raw reads from 101L and 101R were tagged and assembled together. Contigs and singletons generated from combined assembly were annotated using TAIR10, NR, tomato and potato databases.

To identify genes encoding enzymes involved in putative withanolide biosynthetic pathway, total reads from 101L and 101R were tagged, pooled and assembled together. This was carried out to identify total genes involved in different steps of withanolide biosynthesis and to analyze their expression in leaf and root tissues. The size distribution of the contigs from combined assembly is shown in Fig. 4A. The total number of contigs (29,182) in combined assembly is significantly higher in comparison to separate leaf (21,445) and root (20,797) assemblies, which suggests that singletons from single assemblies were incorporated in combined assembly of 101L and 101R. BLAST search of assembled sequences were carried out against the NR, TAIR10, tomato and potato genome databases (Fig. 4B). Annotation summary of combined assembly using each database is provided in Table S11. Of the total, 49,730 unigenes were annotated common and 4694, 50, 2912 and 2348 unigenes annotated uniquely in NR, TAIR10 tomato and potato databases respectively (Fig. 4B).

### Identification of Terpenoid Backbone Biosynthesis Genes

**Figure 5:**
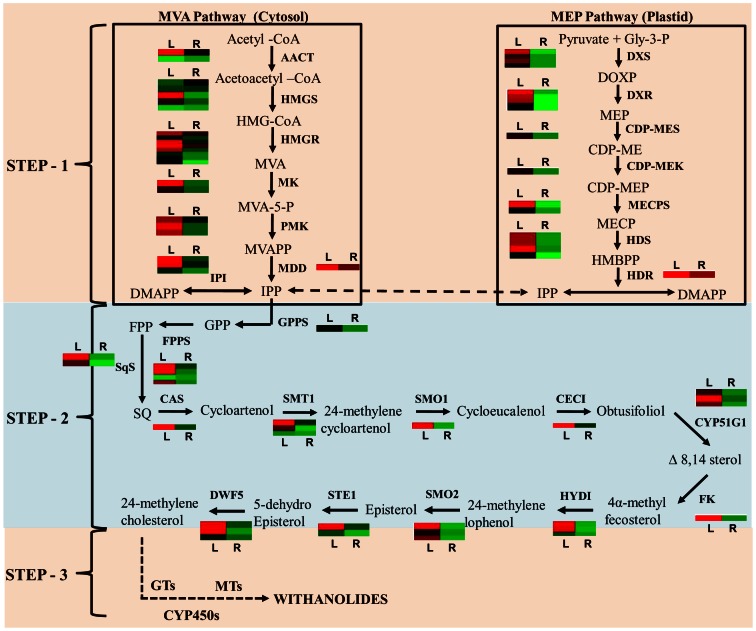
Putative pathway for withanolide biosynthesis in *Withania somnifera*. All the enzymes found in this study related to different steps are shown between the reactions catalyzed. Expression of different contigs related to these enzymes in leaf and root is shown by heatmap. Broken arrow in Step 3 represents putative steps involving CYP450s, GTs and MTs. Names of the enzymes identified are provided in Table 4.

Precursor molecules for withanolides biosynthesis belong to the terpenoid backbone, which utilizes isoprenoids synthesized (Step 1; Fig. 5) via mevalonate as well as MEP pathway [17]. These isoprenoids shares a common pathway from geranyl diphosphate to 24-methylene cholesterol (Step 2; Fig. 5). Annotations from single as well as combined assemblies were used to identify genes encoding enzymes involved in different steps of terpenoid backbone biosynthesis. In combined assembly annotation, contigs and singletons were counted separately (Table 4). In total, Step 1 involves 14 reactions (MVA and MEP) and Step 2 which led to synthesis of 24-methylene cholesterol from IPP requires 13 reactions. All of the genes encoding these enzymes from Step 1 and Step 2 were present in our transcriptome of the *W. somnifera* leaf and root tissues (Fig. 5). In most cases, more than one unigenes were assigned to the same enzyme (Table 4). Such unigenes may represent different fragments of a single transcript, different members of a gene family, or both. Contigs obtained from combined assembly were used to analyze expression pattern of each gene because of their presence in both the tissues. Differential expression analysis suggests that most of the genes involved in triterpenoid biosynthesis show more expression in leaf as compared to root. Similar observation have been made for *Withania* HMGR, FPPS, DXS and DXR in previous studies [11], [17], [22].

**Table 4:** Numbers of unigenes encoding enzymes involved in triterpenoid biosynthesis.

Enzymes	EC	Abbreviation	101L	101R	Combine assembly	
					Contigs	Singletons
**Step 1**						
***MVA pathway***						
Acetyl CoA acetyltransferase	2.3.1.9	AACT	1	4	2	3
Hydroxymethyl glutaryl CoA synthase	2.3.3.10	HMGS	7	4	5	1
3-hydroxy-3-methylglutaryl-coenzymeA reductase	1.1.1.34	HMGR	8	9	8	12
Mevalonate kinase	2.7.1.36	MK	6	1	2	1
Phosphomevalonate kinase	2.7.4.2	PMK	12	15	3	20
Mevalonate diphosphosphate decarboxylase	4.1.1.33	MDD	3	6	1	1
IPP diphosphate isomerase 1	5.3.3.2	IPI	4	2		0
***MEP pathway***						
1-deoxy-D-xylulose-5-phosphate synthase	2.2.1.7	DXS	6	11	4	2
1-deoxy-D-xylulose-5-phosphate reductoisomerase	1.1.1.267	DXR	2	5	5	2
2-C-methyl-D-erythritol4-phosphate cytidylyl transferase	2.7.7.60	CDP-MES	4	2	1	2
4-diphosphocytidyl-2-C-methyl-D-erythritol kinase	2.7.1.148	CDP-MEK	6	1	1	6
2-C-methyl-D-erythritol2,4-cyclodiphosphate synthase	4.6.1.12	MECPS	1	1	2	0
4-hydroxy-3-methylbut-2-enyldiphosphate synthase	1.17.7.1	HDS	3	4	3	0
4-hydroxy-3-methylbut-2-enyldiphosphate reductase	1.17.1.2	HDR	1	1	1	1
**Step 2**						
Geranyl diphosphate Synthase	2.5.1.1	GPPS	2	9	1	6
Farnesyl diphosphate Synthase	2.5.1.10	FPPS	3	6	4	5
Geranylgeranyl diphosphate synthase	2.5.1.29	GGPS	3	3	3	3
Squalene Synthase	2.5.1.21	SQS	4	2	2	4
Squalene Monooxygenase/epoxidase	1.14.99.7	SQE	6	2	1	3
Cycloartenol Synthase	5.4.99.8	CAS	3	9	1	5
Cycloartenol C-24 methyltransferase	2.1.1.41	SMT1	4	5	3	2
Sterol-4α-methyl oxidase 1	1.14.13.72	SMO1	4	4	2	2
Cycloeucalenol cycloisomerase	5.5.1.9	CECI	1	1	1	1
obtusifoliol 14-demethylase	1.14.13.70	CYP51G1	6	5	3	3
Δ14-sterol reductase	1.3.1.70	FK	3	2	1	8
C-7,8 sterol isomerase	5.3.3.5	HYD1	2	1	3	0
Sterol-4α-methyl oxidase 2	1.14.13.72	SMO2	4	6	3	7
C-5 sterol desaturase	1.14.21.6	STE1	1	1	2	2
Sterol Δ7 reductase	1.3.1.21	DWF5	3	4	3	5

### Genes Related to Secondary Modifications and Biosynthesis of Different Withanolides

**Figure 6:**
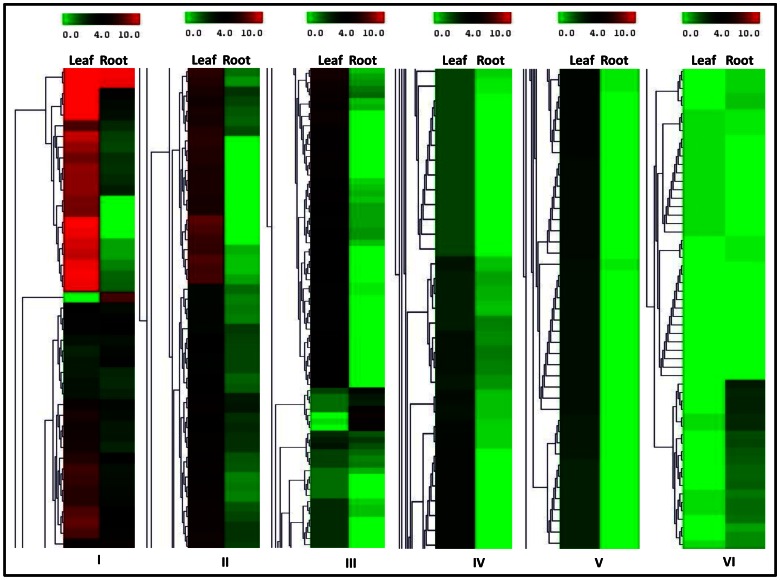
Clusters containing cytochrome P450 with their differential expression in leaf and root. Two columns represent leaf and root, while each row represents contigs encoding different members of CYP450 gene family (Table S12). Clustering was carried out with log_2_tpm value of each contig in leaf and root transcriptome to visualize differential expression.

In *Withania*, leaf and root tissues differ in synthesis of specific withanlodies, as leaf accumulates mainly Withaferin A whereas root tissue accumulates withanolide A [11], [12], [14]. It has been suggested that putative candidate genes involved in Step 3 (Fig. 5) of withanolide biosynthesis, are mainly cytochrome P450s, glycosyltransferases (GTs) and methyltransferases (MTs), which may provide such tissue-specific synthesis and accumulation of withanolide. Through annotation using different databases, 315, 195 and 363 unigenes were identified as members of CYP450, GT and MT gene families respectively (Table 5). Out of all the members, a large number of unigenes were present in specific tissues (Table 5). In addition to specificity, a considerable numbers of unigenes from these families showed significant differential expression between leaf and root tissues (Fig. 6; Fig. S1 and S2; Table S12). Differential expression, as observed for these genes families, may be one of the reasons for differential accumulation of specific withanolides in leaf and root. Apart from these gene families, a total of 4168 unigenes were annotated as transcription factors in our transcriptome data (Table 5; Table S12), out of the total number of transcription factors, 86 and 19unigenes were unique to leaf and root transcriptomes respectively. Transcription factors might be the key regulators for expression of withanolide specific genes in different tissues.

**Table 5:** Contigs encoding various gene families annotated against different databases.

Enzyme Family	Total Contigs	Differentially expressed contigs	Unique in 101L		Unique in 101R
**Cytochrome P450**	305	137		12	36
**Glycosyltransferase**	195	94		7	8
**Methyltransferase**	363	173		11	9
**Transcription factors**	4168	2037		86	19

### Validation of Expression of Differentially Expressed CYP450

**Figure 7:**
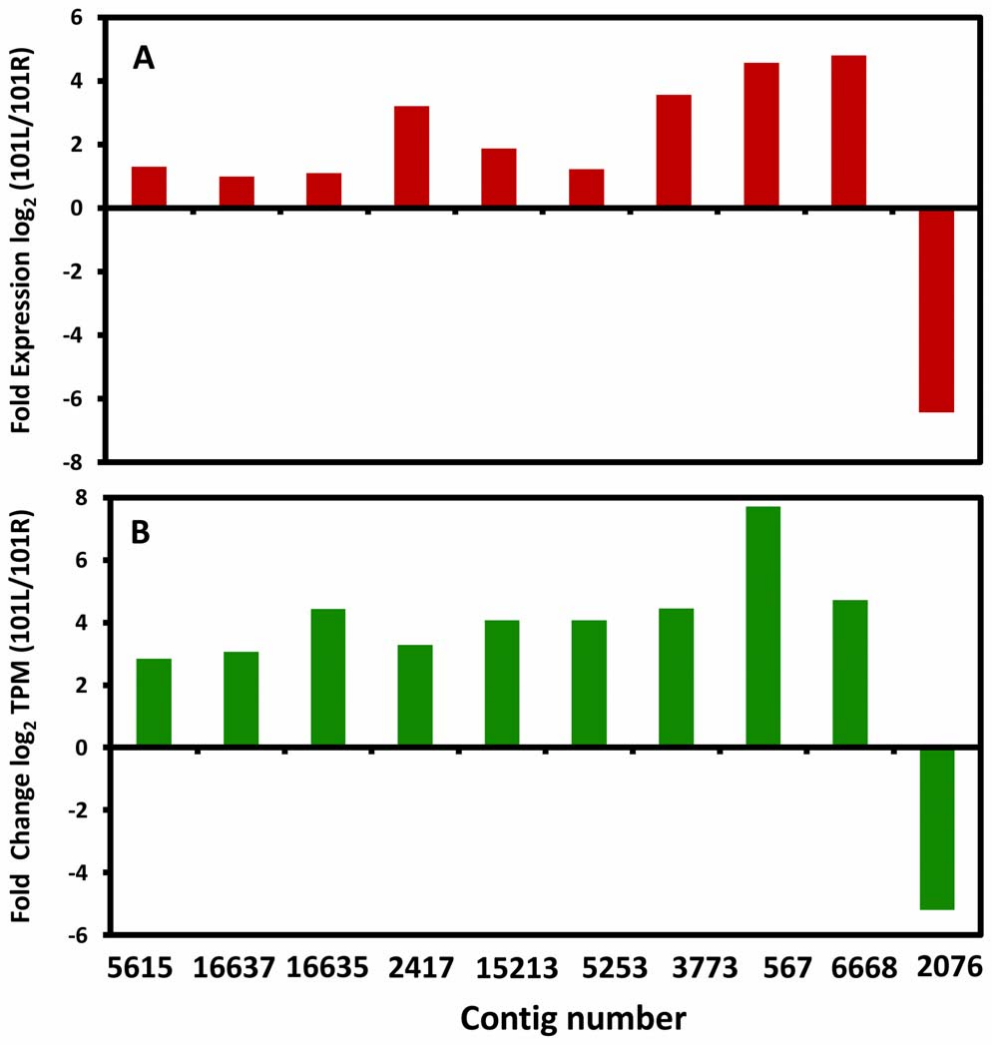
Validation of differentially expressed CYP450 genes in 101L and 101R. Quantitative Real time PCR (A) of selected differentially expressed CYP450s was carried out using total RNA isolated from leaf and root tissues. Digital gene expression (B) of CYP450s used for validating through quantitative Real time PCR.

As there was no information about several steps involved in withanolide biosynthesis, there was need to identify putative genes involved in these steps. Analysis of transcriptome data from 101L and 101R results in identification of genes encoding members of different gene families which might be involved in withanolide biosynthesis. To validate differential expression of these identified genes, we selected 10 CYP450s for validation of expression through quantitative real time PCR (qRT-PCR). All these genes showed differential expression (up and down-regulation) in 101L in comparison to 101R (Fig. 7A). The differential expression observed was similar to digital gene expression results obtained through TPM values (Fig. 7B). Validation of expression of selected genes through qRT-PCR suggests that differentially expressed genes identified through digital gene expression analysis might be differentially expressed between 101L and 101R.

### Identification of SSR Markers

SSRs markers are used in genetic breeding applications of plants and were highly abundant in the 454 transcriptome sequences. Total 21,445 contigs and 68,103 singletons from 101L and 20,797 contigs and 94,017 singletons from 101R were searched for SSR identification. Out of those, 683 contigs and 1684 singletons containing 729 and 1824 total SSR sequences respectively from 101L library were identified. In 101R, 653 contigs and 2283 singletons containing 703 and 2559 total SSR sequences were identified (Table 6). Although following the criteria used to identify these SSRs, tri-nucleotide repeats were the most abundant (320 in 101L and 328 in 101R) and penta-nucleotides were least (3 in 101L and 7 in 101R) in contigs of each library. SSR markers were divided into two groups, perfect SSR markers (only one single repeat motif such as ‘AGC’) and compound SSR markers (composed of two or more SSR markers separated by 100 base pairs). In 101L, a total of 59 compound SSRs from contigs and 142 compound SSRs from singletons and total 74 compound SSR markers from contigs and 198 compound SSRs from singletons were identified in 101R.

**Table 6:** Statistics of SSRs identified from leaf and root transcriptome data.

	101L		101R	
	Contigs	Singleton	Contigs	Singleton
Total number of sequences examined	21445	68103	20797	94017
Total size of examined bases	12668800	19112521	11088108	24408406
Total number of identified SSRs	729	1824	703	2559
Number of SSR containing sequences	683	1684	653	2283
Sequences containing more than one SSR	24	99	28	166
SSRs present in compound formation	86	266	95	461
Di-nucleotide repeat	175	541	177	647
Tri-nucleotide repeat	320	403	328	639
Tetra-nucleotide repeat	8	23	7	24
Penta-nucleotide repeat	3	14	7	15
Hexa-nucleotide repeat	11	26	15	20

## Discussion

Withanolides, secondary metabolites of *W. somnifera,* are synthesized via metabolic divergence from the sterol pathway [16]. Leaves and roots of this plant are considered to have Ginseng-like healthful properties of the herb have been popularized as Indian ginseng. Despite of having medicinal value, there is very little information available for biosynthesis of withanolides from this plant. Transcriptome sequencing through 454 pyrosequencing is regarded as a prime choice for novel gene discovery in non-model organisms and many studies have used de novo assembly of transcriptome data to produce and characterize genome-level resources for non-model organisms [32], [33], [34]. In this study, we used 454 GS-FLX Titanium platform to establish leaf and root transcriptome of *Withania* and carried out de novo assembly and annotation (Table 1; Fig. 1 and 2) to identify putative genes responsible for withanolide biosynthesis.

In our study for leaf and root tissues, 8,34,068 and 721755 HQ reads generated a total number of 89,548 and 1,14,814 unigenes (Table 1) in 101L and 101R respectively. Out of total unigenes, 47,885 (101L) and 54,123 (101R) could be annotated using TAIR10, NR, tomato and potato databases (Fig. 2). This data is in accordance with transcriptome data generated from other medicinal plants. In *Artemisia annua*, 454 pyrosequencing of the glandular trichomes yielded 4,06,044 reads and pyrosequencing of Chinese medicinal plant *Epimedium sagittatum* was developed with 2,17,380 reads assembled into 17,231 contigs and 59,228 singletons [35], [36]. All these databases have helped in identifying genes involved in biosynthesis of medicinal molecules from these plants.

The best hit for each unigene queried against the TAIR10 database was utilized to assign functional GO annotation in terms of biological process, molecular function and cellular component groups. The large number of diverse GO assignments to unigenes highlights the diversity of genes likely represented in *Withania* leaf and root transcriptome data. Mapping these unigenes onto KEGG, we had identified large number of unigenes involved in biosynthesis of various secondary metabolites (Table 3). On the basis of the annotation, we found the genes encoding all of the enzymes involved in biosynthesis of triterpenoid backbone (including MVA and MEP pathways) up to 24-methylene cholesterol (Fig. 5; Table 4), which is considered to be precursor for withanolide biosynthesis [11]. Some of contigs related to enzymes involved in these steps also showed differential expression (Fig. 5).

After biosynthesis of 24-methylene cholesterol, specific enzymes play role in secondary conversion with respect to transfer of different moieties or oxidation/reduction reactions leading to biosynthesis of specific withanolides in tissue-specific manner [16]. It has been suggested that, gene families, which play major role in secondary conversion steps (Step 3; Fig.5) of specific withanolide biosynthesis, are cytochrome P450s (CYP450s), glycosyltransferases (GTs) and methyltransferases (MTs). Since the number of 454 pyrosequencing reads in each contig is directly proportional to the abundance of specific cDNAs in the library, quantification of reads for specific contig provides an accurate measure of the relative expression level of selected transcripts. This has helped us to study differential expression of genes related to secondary conversion and their putative role in tissue-specific withanolide biosynthesis. CYP450s may catalyze hydroxylation reactions to synthesize various withanolides (withanolide A, withanolide D, withaferin A and withanone) from 24-methylene cholesterol as these enzymes catalyze most of the oxidation/hydroxylation steps in plant secondary metabolism [37], [38], [39]. The CYP450s constitute one of the biggest gene families in plant genomes (accounting for more than 1% of the total gene annotations) in each plant species and are generally involved in the biosynthesis of terpenoids, sterols, lignins, hormones, fatty acids, pigments, and phytoalexins in plants [40]. In our analysis, we discovered total of 305 contigs encoding CYP450s, out of which 12 and 36 unique CYPs for leaf and root tissues were present respectively (Table 5). These might be involved in differential activities in these tissues including withanolide biosynthesis. In *Withania*, withanosides and sitoindosides are the glycosylated form of steroidal lactones and may be synthesized through action of GTs [18], [41], [42]. In our study, we observed number of unique GTs for leaf and root tissues (Table 5). Apart from unique CYPs and GTs in leaf and root, a large number of members from these genes families showed differential expression in root and leaf (Fig. 6; Fig. S1). The study has also identified large number of MTs and transcription factors, some of them show differential expression in leaf and root tissues (Fig. S2; Table S12). Unigenes identified in this study will be valuable for elucidation of the withanolide biosynthesis pathway and to the exploration of the molecular mechanism underlying the biosynthesis of specific withanolides in root and leaf tissues.

In addition to gene identification for pathway elucidation, we also identified molecular markers using transcriptome data from leaf and root (Table 6). Similar to our analysis, next-generation sequencing technologies have been used for development of molecular markers in non-model organisms [43], [44], [45]. In our analysis, the frequency of tri-nucleotide repeats is highest in the distribution of EST-SSRs as has also been observed in other studies [46]. In conclusion, our study is the first to establish high-quality transcriptome database for *Withania* using NGS technology. Enzymes involved in biosynthesis of triterpenoid as well as putatively involved in withanolide biosynthesis have been identified. The discovery of genes associated with biosynthetic pathways as well differentially expressing CYPs, GTs, MTs and transcription factors provides an important resource in *Withania* research. The information will help in developing strategies of metabolic engineering to increase the production of specific withanolides. Additionally, the detection of EST-SSRs will assist in the dissection of complex genetic background of *Withania* especially in relation to distinct chemotypes. Our transcriptome data provides usefulness of transcriptome analysis based on NGS technology to further research and ongoing development on *Withania*.

## Supporting Information

Figure S1 Clusters containing methyltransferases (MTs) with their differential expression in leaf and root. Two columns represent leaf and root, while each row represents contigs encoding different members of MT gene family (Table S12). Clustering was carried out with log_2_tpm value of each contig in leaf and root transcriptome to visualize differential expression.
(TIF)

Figure S2 Clusters containing glycosyltransferases (GTs) with their differential expression in leaf and root. Two columns represent leaf and root, while each row represents contigs encoding different members of GT gene family (Table S12). Clustering was carried out with log_2_tpm value of each contig in leaf and root transcriptome to visualize differential expression.
(TIF)

Table S1 List of different contigs encoding CYP450 and primers used for validation of expression through qRT-PCR.
(DOC)

Table S2 Annotation of *Withania* leaf transcriptome (101L) using NR database.
(XLS)

Table S3 Annotation of *Withania* leaf transcriptome (101L) using TAIR10.
(XLS)

Table S4 Annotation of *Withania* leaf transcriptome (101L) using tomato genome database.
(XLS)

Table S5 Annotation of *Withania* leaf transcriptome (101L) using potato genome database.
(XLS)

Table S6 Annotation of *Withania* root transcriptome (101R) using NR database.
(XLS)

Table S7 Annotation of *Withania* root transcriptome (101R) using TAIR10.
(XLS)

Table S8 Annotation of *Withania* root transcriptome (101R) using tomato genome database.
(XLS)

Table S9 Annotation of *Withania* root transcriptome (101R) using potato genome database.
(XLS)

Table S10 Percent of unigenes involved in various GO functional categories.
(XLS)

Table S11 Annotation of 101L and 101R combined assembly using NR, TAIR10, tomato and potato genome databases.
(XLS)

Table S12 Contigs and their fold change in leaf (101L) and root (101R) transcriptomes showing homology to different gene families.
(XLS)
