# Supplementary figures and images for: De Novo Assembly, Functional Annotation and Comparative Analysis of Withania somnifera Leaf and Root Transcriptomes to Identify Putative Genes Involved in the Withanolides Biosynthesis
Source: PLoS One. 2013 May 8;8(5):e62714. doi: 10.1371/journal.pone.0062714 (PMC3648579; doi:10.1371/journal.pone.0062714)

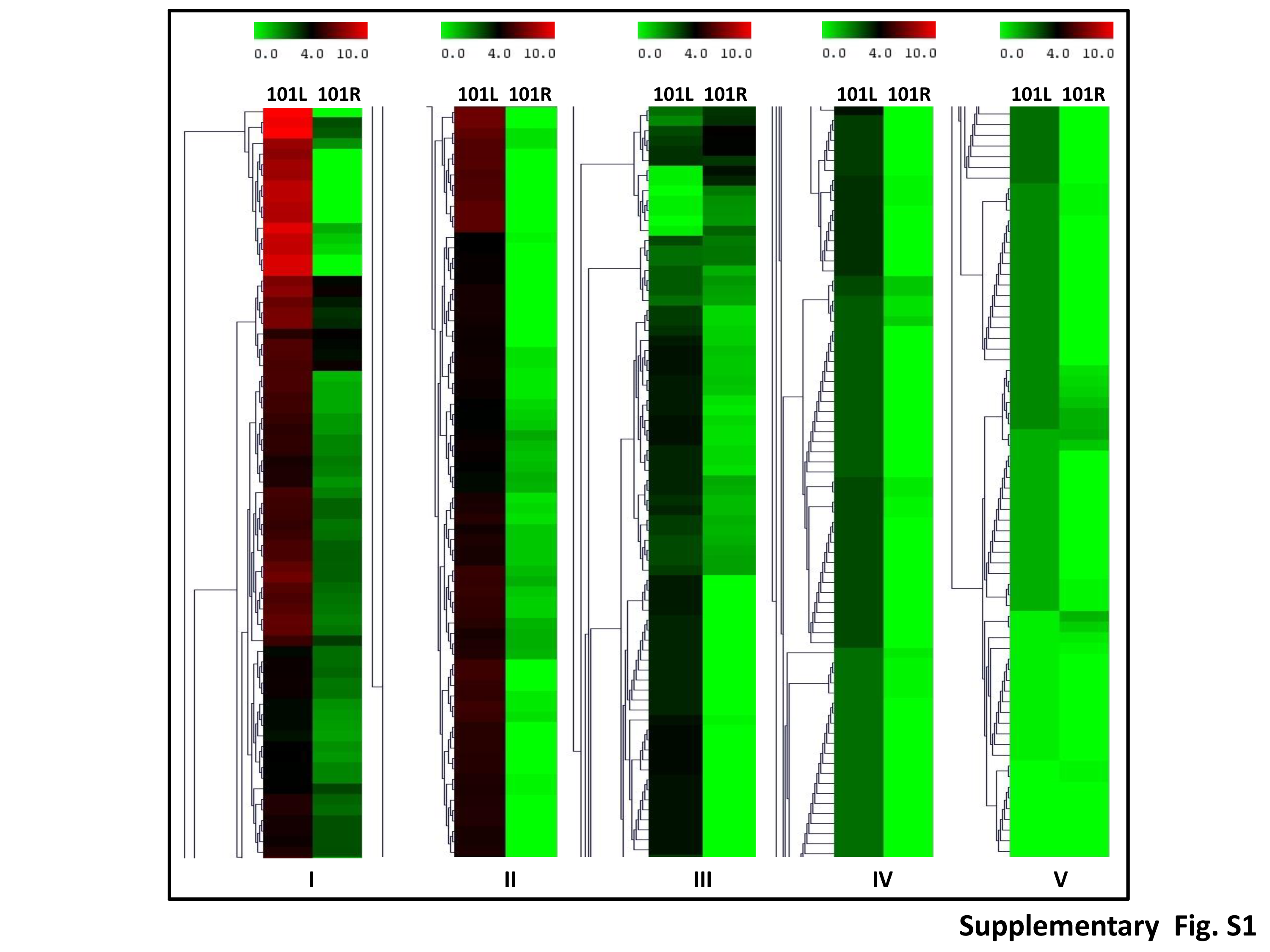

Supplement: Figure S1 — Clusters containing methyltransferases (MTs) with their differential expression in leaf and root. Two columns represent leaf and root, while each row represents contigs encoding different members of MT gene family (Table S12). Clustering was carried out with log2tpm value of each contig in leaf and root transcriptome to visualize differential expression. (TIF) [file pone.0062714.s001.tif]

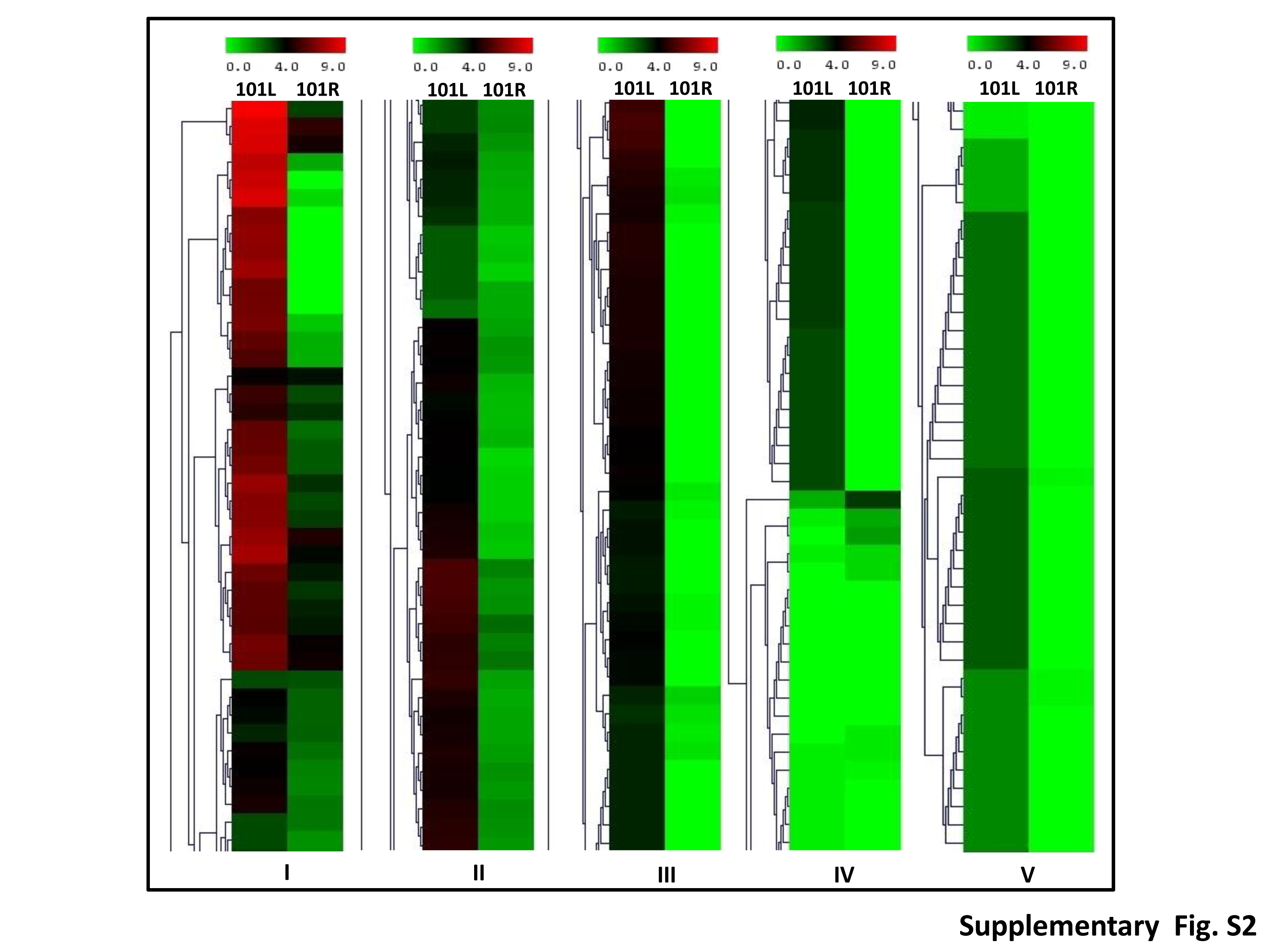

Supplement: Figure S2 — Clusters containing glycosyltransferases (GTs) with their differential expression in leaf and root. Two columns represent leaf and root, while each row represents contigs encoding different members of GT gene family (Table S12). Clustering was carried out with log2tpm value of each contig in leaf and root transcriptome to visualize differential expression. (TIF) [file pone.0062714.s002.tif]
