# Supplementary material for: De Novo Assembly, Functional Annotation and Comparative Analysis of Withania somnifera Leaf and Root Transcriptomes to Identify Putative Genes Involved in the Withanolides Biosynthesis
Source: PLoS One. 2013 May 8;8(5):e62714. doi: 10.1371/journal.pone.0062714 (PMC3648579; doi:10.1371/journal.pone.0062714)
Supplement: Table S1 — List of different contigs encoding CYP450 and primers used for validation of expression through qRT-PCR. (DOC) [file pone.0062714.s003.doc]

| **Contig No. No. No.** | **Forward Primer (5' to 3')** | **Reverse Primer (5' to 3')** |
| --- | --- | --- |
| **5615** | AGGATTAGGCCAAGGGAAAGCAGAGA | CTTCCATTGATTCTCGAGCAACTAGCA |
| **16637** | TGGTGTGGACTTGTCCGAGTGCTTG | TCAAACCCAAGTAAACTTTCAATGAACC |
| **2417** | TCGGAACCCAGACTGGTACAGCTGAA | TCTTCATCATCAGCCGCATAATCATCCA |
| **5253** | AGAAGAGCTCAAGTTGCAGATGTGCTC | CTGCTCTATCCCATACCTCCGGAGAAT |
| **15213** | TCATGGTTAAGGACCCTGAACATAGCTC | ATGTCAACTCAGTGTCAAGCCAAAGAAC |
| **16635** | AACGAGGCTGCAGTCTTATCGGAC | TAAGAGGCAAATCGAGACACTTGTTCCA |
| **3773** | TGGAAGCAGCAGaGAAAGCTCGCAAG | TTGCTGACCAATTTGGCTGCTCGT |
| **567** | ATGAAGTGCACGGATTAGCCCAAGGAA | TCGAGGAATTAGTAGTGGAACTGGTGGA |
| **6668** | CGACATACCTATGGATGCTAACGTGGA | GTCAAACTTATCCGGCTCGGACCATA |
| **2076** | TGTCTGTTCTCCTGAGCTTGCT | TGAGGTCAAGTAGCCATTGCTAAC |

**Supplementary Table S1:** List of different contigs encoding CYP450 and primers used for validation of expression through qRT-PCR
